# Understanding the Genetic and Molecular Basis of Familial Hypertrophic Cardiomyopathy and the Current Trends in Gene Therapy for Its Management

**DOI:** 10.7759/cureus.17548

**Published:** 2021-08-29

**Authors:** Roshini Pradeep, Aqsa Akram, Matthew C Proute, Nageshwar R Kothur, Petros Georgiou, Tatsiana Serhiyenia, Wangpan Shi, Mina E Kerolos, Jihan A Mostafa

**Affiliations:** 1 Internal Medicine, California Institute of Behavioral Neurosciences & Psychology, Fairfield, USA; 2 Family Medicine, California Institute of Behavioral Neurosciences & Psychology, Fairfield, USA; 3 Pathology, California Institute of Behavioral Neurosciences & Psychology, Fairfield, USA; 4 Medicine, California Institute of Behavioral Neurosciences & Psychology, Fairfield, USA; 5 Psychiatry/Cognitive Behavioural Psychotherapy, California Institute of Behavioral Neurosciences & Psychology, Fairfield, USA

**Keywords:** cardiology, hypertrophic cardiomyopathy, genetics, epigenetics, signal transduction, pathophysiology, gene therapy

## Abstract

Hypertrophic cardiomyopathy (HCM) is a genetically acquired disease of cardiac myocytes. Studies show that 70% of this disease is a result of different mutations in various sarcomere genes. This review aims to discuss several genetic mutations, epigenetic factors, and signal transduction pathways leading to the development of HCM. In addition, this article elaborates on recent advances in gene therapies and their implications for managing this condition. We start by discussing the founding mutations in HCM and their effect on power stroke generation. The less explored field of epigenetics including methylation, acetylation, and the role of different micro RNAs in the development of cardiac muscle hypertrophy has been highlighted in this article. The signal transduction pathways that lead to gene transcription, which in turn lead to increased protein synthesis of cardiac muscle fibers are elaborated. Finally, the microscopic events leading to the pathophysiologic macro events of cardiac failure, and the current experimental trials of gene therapy models, and the clustered regularly interspaced short palindromic repeats (CRISPR) type 2 system proteins, are discussed. We have concluded our discussion by emphasizing the need for more studies on epigenomics and experimental designs for gene therapy in HCM patients. This review focuses on the process of HCM from initial mutation to the development of phenotypic expression and various points of intervention in cardiac myocardial hypertrophy development.

## Introduction and background

Hypertrophic cardiomyopathy (HCM), with its mortality of six percent annually, is an autosomal dominant disease due to sarcomeric-related gene mutations. Though its prevalence as of date is one in 600, the genetics behind this disease is minimally explored. HCM is heterogeneous with variable clinical expression and outcome within the same family. Though genetic mutation is the predisposing factor to the development of the disease, phenotype variability can be observed due to mechanisms underlying incomplete penetrance and variable expressivity. Various genetic and non-genetic modifiers can be responsible for developing histopathologic changes, including myocyte hypertrophy and disarray, fibrosis, and small vessel disease [1].

The genetic mutation involved can be classified into mutations in eight core genes and various emerging genes. Among these, 75% of the genotyped cases have been associated with mutations in the myosin binding protein C (*MYBPC3*) and β-myosin heavy chain (*MYH-7*) [1,2]. Other than the causative genes, various 'modifier genes' can influence disease expression and alter the disease course in the susceptible population. The positive correlation of angiotensin-converting enzyme (ACE) polymorphism with HCM has been well studied [3]. In addition to this, various modifiable risk factors such as hypertension, physical activity, and non-modifiable risk factors such as gender have been studied to explore further the penetrance and severity of phenotypic expression in a population with genetic mutation developed HCM [4]. Along with the genetic, demographic, environmental, and comorbid factors, it is important to note that epigenetics has its place in the pathophysiology of this disease. 

A modification of gene expression without alteration of the genetic code itself is called epigenetics. In simple terms, It refers to external modifiers, which play a role in turning the DNA 'on' and 'off'. This change in gene expression can be caused by various mechanisms, including DNA methylation/demethylation, histone modification, non-coding RNAs (ncRNA), and post-translational regulation [1]. In individuals with strong genetic history for HCM, phenotypic expression of the disease is determined by a modification of the epigenome. To illustrate this, methylation of CpG islands of cardiac troponin T leads to genetic instability, which further leads to the deamination of this region, and this causes mutations that later predispose to HCM [5]. CpG islands are regions with a high frequency of CpG sites, which are sites where cytosine (C) lies next to guanine (G).

On the contrary, a positive correlation has been established as a result of the interaction of brahma-related gene (Brg-1) with histone deacetylases and poly (adenosine diphosphate (ADP)-ribose) polymerase-1 (PARP-1), which decreases the susceptibility of HCM in this population [6]. It is also noteworthy that ncRNA that includes long non-coding RNA (lncRNA) and microRNA (miRNA) are constantly changing depending on the disease stage, which can be a potential severity marker in the disease course [7]. In addition to this, phosphorylation of certain DNA segments has been shown to compensate for the pathological consequence of HCM mutations [8].

This article will explore the genetics and environmental factors leading to the development of HCM, with particular emphasis on the epigenetic factors that can alter the course of the disease. Also, we will discuss the various treatment strategies based on ongoing clinical trials targeting the modifier genes.

## Review

Genetic mutations and their effects on hypertrophic cardiomyopathy

The data suggests that over 450 mutations, which include 20 sarcomeric and myofilament-related proteins, associate with HCM [9]. Various studies have shown that sarcomeric genes, including *MYH7* and *MYBPC3*, comprise most mutations [10]. The identification of the genetic cause of HCM dates back to 1990 when a large family presenting with sudden death secondary to HCM had specific sarcomeric mutations [11,12]. Further evidence has indicated multiple such mutations in various sarcomeric proteins.

The missense variant (Arg453Cys) in the *MYH7* non-rearranged segment is a definitive cause of the disease [13]. Although β myosin heavy chain and *MYBPC3* constitutes 50% of the mutations, the other mutations in HCM include *TNNT2*, α tropomyosin (*TPM1*), myosin essential light chain (*MYL3*), myosin regulatory light chain (*MYL2*), and actin alpha cardiac muscle 1 (*ACTC1*) [14,15,16]. Furthermore, the clinical course of HCM defect is determined by a combination of *MYBPC3* founding mutations with another HCM defect [17]. The primary mechanism behind this is that *MYH7* mutations lead to abnormal actin-myosin interactions, diminishing sarcomeric force production [18]. Thus, hypertrophic remodeling is a compensatory response to diminished cardiac performance [19]. Also, the mutations might affect the calcium sensitivity of the troponin complex, leading to the generation of the power stroke [20]. The core genes associated with sarcomeric HCM are shown in Table 1.

**Table 1:** Core gene mutations involved in the development of hypertrophic cardiomyopathy

Core Genes	Sarcomere proteins	Frequency	Author names
Thick Myofilament			
MYBPC3	Myosin binding Protein C	40%	Barefield D et al. 2014 [21]
MYH7	β-myosin heavy chain	40%	Hershkovltz T et al. 2019 [22]
MYL2	Myosin light chain 2	<1%	ManIvannan SN et al. 2020 [23]
MYL3	Myosin light chain 3	<1%	Kabaeva Z et al. 2002 [24]
Thin Myofilament			
ACTC1	Cardiac α-actin	<1%	Yang QL et al. 2019 [25]
TPM1	α – Tropomyosin	<1%	Jongbloed RJ et al. 2003 [26]
TNNT2	Cardiac troponin T	10%	Pasquale F et al. 2012 [27]
TNNI3	Cardiac troponin I	<5%	van den Wijngaard A et al. 2011 [28]

The Initial set of changes induced by the mutations includes altered transcriptional rate and translational efficiency, and it also involves a change in sarcomere protein and its function [29]. One of the mechanisms involved is a gain of stop codon prematurely terminates the transcription, which is later degraded by the decay-inducing complex, leading to decreased protein levels. These characteristics are predominantly observed during mutations in the *MYBPC3* gene [29,30]. Another type of mutation observed is a missense mutation that can lead to structural changes in encoded protein, which can further cause reduced efficiency of sarcomere assembly [31]. Furthermore, it has been studied that variable expression of these mutant proteins and their incorporation into sarcomere and myofilaments is responsible for variable functional defects observed [32]. Therefore, these mutant protein incorporations might further explain the variable phenotypic expression of HCM. Though the mutation is the primary cause of the HCM, cardiac hypertrophy results from intermediary molecular events and many pathways [33]. This pathophysiology is similar to that of other forms of cardiac hypertrophy, such as cardiac pressure overload. These pathways include calcineurin activation, transforming growth factor β pathway, mitogen-activated protein kinases, ncRNA, and epigenetic factors [34]. All these together result in morphologic and histologic phenotypes recognized as HCM. 

Role of epigenetic factors in the phenotypic expression of hypertrophic cardiomyopathy

The *MYBPC3* has been reported to have the second-highest number of causal mutations for familial HCM. Evidence suggests that a higher methylation level of exonic CpGs in the *MYBPC3* gene may lead to deamination of methylated CpGs, contributing to mutation development [1,35]. The frequency of this mutation development is variable among individual human genes [35]. This mechanism of many genetic mutations distributed throughout each gene can be attributed to the deamination of highly methylated CpG sites within cardiac genes. In addition, it has been observed that fairly common HCM mutation due to G-to-T transversion can be caused by carcinogens such as benzo(a)pyrene diol and acrolein binding to methylated CpG sites [35,36].

**Figure 1:**
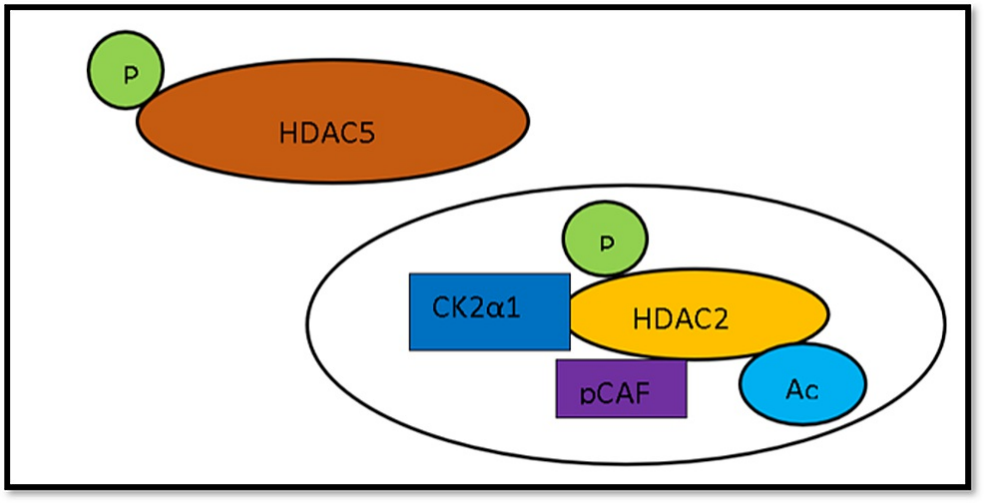
Downstream effects of phosphorylated histone deacetylase-5 Phosphorylated *HDAC5* leading to the shuttling of CK2α 1 into the nucleus that phosphorylates *HDAC2*, CBP-associated factor (pCAF ) binds to *HDAC2*, inducing acetylation. Further downstream, this leads to hypertrophy of the myocardium. Modified from Figure 7 in Eom GH et al. [39] HDAC5: histone deacetylase-5; HDAC2: histone deacetylase-2; pCAF: CBP-associated factor (CBP: CREB-binding protein; CREB: cyclic-AMP response element-binding protein; AMP: adenosine monophosphate)

The histones are the most critical players in maintaining chromatin in a silenced or active state by DNA interaction [37-39]. Histone deacetylases 5 (*HDAC5*) inhibits histone deacetylases 2 (*HDAC2*) by deacetylation. When the myocardium is stimulated by hypertrophic stress, phosphorylation of *HDAC5* occurs, as shown in Figure 1. This phosphorylated *HDAC5* gets transported to the cytoplasm from the nucleus [40]. Additionally, casein kinase 2α1 (CK2α 1) is shuttled into the nucleus and phosphorylates *HDAC2*, and CBP-associated factor (pCAF ) binds to *HDAC2*, inducing acetylation (CBP: CREB-binding protein; CREB: cyclic-AMP response element-binding protein; AMP: adenosine monophosphate). As a result, activation of *HDAC2* leads to hypertrophy of the myocardium [39]. Thus, this study emphasized that histone deacetylases play an essential role in cardiac hypertrophy development.

As per various studies, it is proven that miRNA levels are constantly changing in different stages of HCM. miRNA is composed of a 22 nucleotide ncRNA. It demonstrates its negative regulation by complementary mRNA silencing [1,41]. Most studied miRNAs in HCM have shown increased pro-fibrotic and pro-hypertrophic miRNAs and decreased miRNAs, inducing opposite effects. It has been observed that miR-1 and miR-133, found in cardiac tissue, physiologically exert anti hypertrophic effects in the heart by targeting multiple hypertrophic signaling molecules [42]. Some of these include angiotensin 2 receptor type 1 (AGTR1), transforming growth factor (TGF) β, and fatty acid-binding protein (FABP) 3 [1,43]. Interestingly, in mouse models of HCM, It has been found that miR-1 and miR-133 in cardiac tissue are downregulated early in the disease phase and even before target gene upregulation [7]. Though the hypothesis holds that augmentation of circulating miR-1 and miR-133 in asymptomatic patients can prove a cutting edge to early diagnosis of HCM, the limitation of harvesting required tissue remains a challenge in vivo [44]. For this reason, recent studies have targeted identifying levels of miRNAs in plasma as they prove to have more clinical value due to their minimally invasive nature, longer half-life, and high stability [1,45]. Derda et al. have identified a specific signature of miRNA, miR-29a, which is exclusively elevated in the plasma of subjects with obstructive HCM [46]. This finding was associated with a robust correlation between miR-29a and the size of the interventricular septum. Another essential type of ncRNA is IncRNA. Known to have no protein-coding function, lncRNA influences gene expression at transcriptional and post-transcriptional levels [1]. Its role in HCM development is through control of chromatin remodeling and combining with the corresponding miRNA. A recent study identified that levels of IncRNA myocardial infarction associated transcript (MIAT) have an inverse correlation with miR-29a expression in HCM. They reported that subjects without fibrosis exhibited high levels of lncRNA-MIAT and reduced levels of miR-29a compared to the subjects with fibrosis [47]. Therefore, it has been hypothesized that IncRNA-MIAT can act as an endogenous miRNA sponge to regulate the expression of miR-29a-3p, proof of how miRNAs can influence gene expression in subjects with HCM. 

Overview of signal transduction pathways involved in cardiac hypertrophy

Cardiac hypertrophy development encompasses a complex set of pathways, leading to phenotypic changes. This complex set of pathways involved in cardiomyocyte hypertrophy includes dozens of ligands, receptors, cytoplasmic signal amplifiers, and transcriptional effectors [48,49]. The various signal transduction molecules include mitogen-activated protein kinase (MAPK), tyrosine kinase, insulin-like growth factor (IGF), TGF, fibroblast growth factor (FGF), c-Jun N-terminal kinase (JNK), and protein kinase C(PKC). These pathways further influence change in gene transcription that leads to changes in protein synthesis, which leads to cardiac hypertrophy [50]. One of the first to provide molecular insight on how extracellular signals travel from cell membrane to nucleus is the calcineurin/nuclear factor of the activated T-cell (NFAT) pathway [51]. It is well understood that a significant downstream effector of cyclic guanosine monophosphate (cGMP) signaling in cardiomyocytes is cGMP-dependent protein kinase-1 (PKG-1). Various rodent studies have shown that NFAT nuclear translocation is reduced by cGMP-induced PKG-1 activation, which plays a significant role in inhibiting cardiac hypertrophy [52]. A recent study has observed interaction between phosphoinositide 3-kinase (PI3K)-Akt signaling pathway, which is known to cause physiological cardiac hypertrophy, and protein kinase C beta (PKCβ) 2, which is known to cause pathologic cardiac hypertrophy. This study involved crossing transgenic mice to observe potential interaction between PI3K and PKCβ pathways. Further observation for expression of PKCβ2, constitutively active PI3K, and dominant-negative PI3K suggested that PI3K can act as an upstream modulator of PKCβ2 and this, in turn, can rescue the pathologic cardiac dysfunction induced by overexpression of PKCβ2 [53]. Further studies on lipid kinases involved in signal-transduction cascades may provide insight into potential intervention targets in preventing heart failure secondary to HCM. Also, multiple research is indicated on understanding various molecular pathways in the development of cardiac hypertrophy to translate this scientific knowledge into potential pharmacotherapies to treat hypertrophic cardiomyopathy. 

Pathophysiology of hypertrophic cardiomyopathy

**Figure 2:**
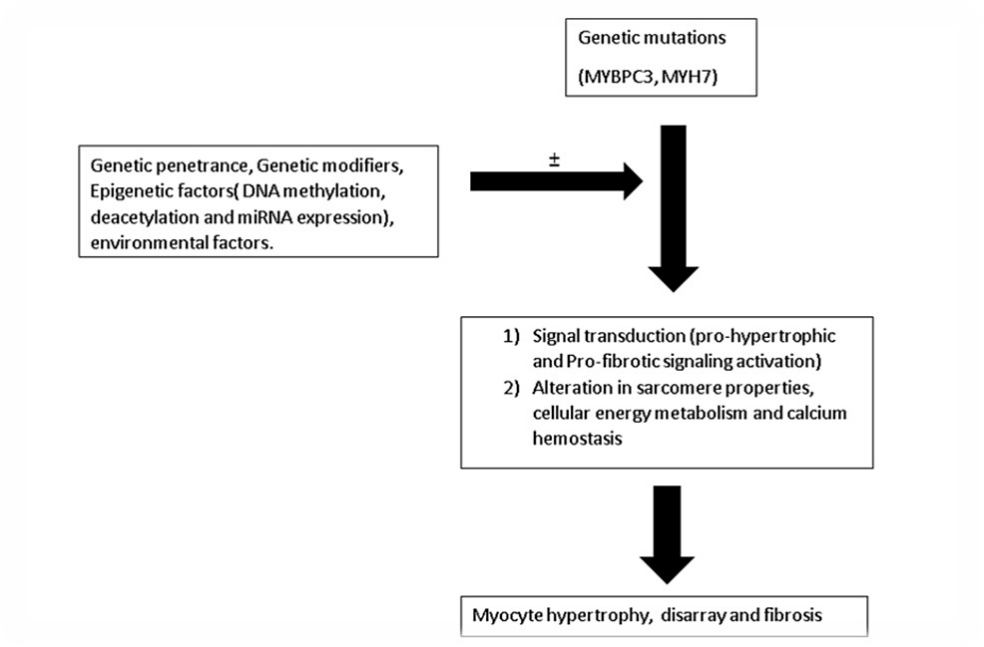
Factors regulating the development of myocardial hypertrophy in individuals with HCM mutation This figure shows that genetic penetrance, genetic modifiers, epigenetic factors (DNA methylation, deacetylation, and miRNA expression), and environmental factors play a regulatory role after the initial mutation and determine the downstream effects, including signal transduction, alteration in sarcomere properties, cellular energy metabolism, and calcium hemostasis. The upregulation of pro-hypertrophic and pro-fibrotic signal transduction pathways further leads to myocyte hypertrophy, disarray, and fibrosis. Figure modified from Wolf CM [57] *MYBPC3: *myosin-binding protein C-3; *MYH7*: myosin Heavy Chain 7; HCM: hypertrophic cardiomyopathy

Myocardial hypertrophy and ventricular dysfunction are the functional pathologies observed in HCM. Hypertrophy results from the interplay of various factors, including microvascular ischemia, energy depletion, and apoptosis of cardiomyocytes. The progressive myocyte loss and fibrous substitution lead to the adverse remodeling of the myocardium [1,11]. These changes further lead to an irreversible stage of overt myocardial dysfunction, leading to severe heart failure and death. In addition, these changes are responsible for the physiopathologic changes of left ventricular hypertrophy, diastolic dysfunction, left ventricular outflow obstruction, and arrhythmias. As a result, people suffering from HCM develop dyspnea, chest pain, syncope, palpitations, and sudden cardiac death [11]. A study by Ho et al. demonstrated that mutant gene carriers of HCM have significantly more fibrosis detected through late gadolinium enhancement compared to normal subjects but less than an overt disease [54]. A similar study by Shirani et al. found that children and young adults with HCM had three to eight times more fibrous tissue than hypertensive or healthy subjects [55]. Additionally, one other study hypothesizes that the disorganized sarcomeric and cellular architecture, neuroendocrine activation, and myocardial ischemia can cause increased mechanical stress and increase in nuclear factor kappa-a transcription factor regulating inflammation. This upregulation leads to increased fibroblast activation, which eventually leads to fibrosis [56]. From the above discussion, studies highlight that the significant transient point between the genotype to the phenotypic presentation of HCM is the histopathologic changes. Hence, the mutations and a complex hierarchy of epigenetics and environmental factors are determinants of the clinical expression of the disease (Figure 2). 

Current developments for gene therapy in hypertrophic cardiomyopathy

HCM is one of the leading causes of sudden cardiac death in individuals in their early 30s. The current treatment strategies of drug-based therapy aim to relieve symptoms, but they do not target the causal genes [58]. Clyde Keller first introduced the idea of gene therapy in 1947 [59]. As of today, more than 2300 clinical trials are approved worldwide on various gene therapies for the cure of hereditary diseases [60]. The adeno-associated viruses (AAVs) are the viral vectors used currently for genetic therapy of cardiac diseases. They belong to the Parvoviridae family with very low pathogenicity in the human host [61]. As of today, AAV9 has proved most efficient for cardiac gene transfer after various trials in mouse and large animal models [62,63]. The sarcoplasmic reticulum calcium ATPase 2a (SERCA2a) gene therapy phase 2 trial has demonstrated that AAV1-mediated gene transfer is safe and feasible in humans but has failed to show beneficial outcomes in this cohort [64,65]. A more recent development is that the CRISPR type-2 system proteins (Cas9) can function as designer nucleases when combined with an engineered single guide RNA, complementary to a genetic locus of interest [66]. The mechanism of action Cas9 is by inducing a double-stranded break that can be resolved by non-homologous end joining [67,68].

An insightful study by Ma et al. focused on a male HCM patient with a familial history of HCM by GAGT-deletion in exon 16 of the *MYBPC3* gene. The author reported that endogenous, germline-specific DNA repair responses led to the successful correction of germline mutations. A mutant paternal allele with double-stranded breaks was repaired using a homologous wild-type maternal gene. This resulted in increased homology-directed repair efficiencies up to 27% among targeted clones. Interestingly, there were no off-target events found [69]. A subsequent study has also shown that AAV-mediated exon skipping with antisense nucleotide or CRISPR/Cas9 at *MYBPC3* mutation hot spots such as exon 25 containing 11% of hotspots remains a valuable therapeutic approach [70]. Though these studies remain controversial as interhomolog recombination has never been reported before, methods such as preimplantation genetic diagnosis can avoid editing human embryos [71]. However, we cannot deny that studies such as these have led us to explore the effect of CRISPR/Cas9 in treating human germline mutation, which is most likely going to be the cutting edge technology in finding a definitive cure for hereditary diseases such as HCM.

## Conclusions

Various studies have explored the different aspects of HCM from the view of genomics, biochemistry, and gene therapies separately. This article aims to bring the molecular knowledge of HCM together, so it becomes easier to connect the dots and hence work from here on. The idea is to explore the mutations associated with HCM and elucidate various genetic and molecular targets for further experimentation. There have been various advances in identifying the methylation of exons, acetylation of multiple enzymes, and various miRNA levels in HCM. However, the full potential of epigenomics in HCM is yet to be discovered. The main limitation of this study is that we have focused on literature that was specific to our discussion. Therefore, certain aspects such as various other discovered genes, molecular mechanisms, and gene therapies, are not discussed here. We recommend further studies specifically on identifying epigenetic modifications and targeted gene therapies. The targeted experimental designs might help us invent new interventional models that can help us on direct management of HCM early on by addressing the cause of the disease. We strongly believe that if further studies can demonstrate successful human trials by targeted genetic and molecular aspects of the disease, we might eventually find definitive management for HCM in the future.
